# The effectiveness of immediate versus delayed tubal flushing with oil-based contrast in women with unexplained infertility (H2Oil-timing study): study protocol of a randomized controlled trial

**DOI:** 10.1186/s12905-023-02385-1

**Published:** 2023-05-06

**Authors:** D. Kamphuis, K. Rosielle, N. van Welie, I. Roest, A. J.C.M. van Dongen, E. A. Brinkhuis, P. Bourdrez, A. Mozes, H. R. Verhoeve, D. P. van der Ham, F. P.J.M. Vrouenraets, J. J. Risseeuw, T. van de Laar, F. Janse, J. E. den Hartog, M. de Hundt, A. B. Hooker, A. G. Huppelschoten, Q. D. Pieterse, M. Y. Bongers, J. Stoker, C. A.M. Koks, C. B. Lambalk, A. Hemingway, W. Li, B. W.J. Mol, K. Dreyer, V. Mijatovic

**Affiliations:** 1 Department of Reproductive Medicine, Amsterdam UMC location Vrije Universiteit Amsterdam, De Boelelaan 1117, 1081HV, Amsterdam, 1081 HV The Netherlands; 2 Amsterdam Reproduction and Development Research Institute, Amsterdam, The Netherlands; 3 Department of Obstetrics and Gynaecology, OLVG, Amsterdam, 1091 AC The Netherlands; 4 Department of Obstetrics and Gynaecology, Máxima Medisch Centrum, Veldhoven, Eindhoven, 4600 DB The Netherlands; 5 Grow research school for Oncology and Reproduction, Maastricht University, Universiteitssingel 40, Maastricht, 6229 ER The Netherlands; 6 Department of Obstetrics and Gynaecology, Ziekenhuis Gelderse Vallei, Ede, 6716 RP The Netherlands; 7 Department of Obstetrics and Gynaecology, Meander Medisch Centrum, Amersfoort, 3813 TZ The Netherlands; 8 Department of Obstetrics and Gynaecology, VieCuri Medisch Centrum, Venlo, 5912 BL The Netherlands; 9 Department of Obstetrics and Gynaecology, Ziekenhuis Amstelland, Amstelveen, 1186 AM The Netherlands; 10 Department of Obstetrics and Gynaecology, Martini Ziekenhuis, Groningen, 9728 NT The Netherlands; 11 Department of Obstetrics and Gynaecology, Zuyderland Medisch Centrum, Heerlen, 6419 PC The Netherlands; 12 Department of Obstetrics and Gynaecology, St. Jansdal Ziekenhuis, Harderwijk, 3844 DG The Netherlands; 13 Department of Obstetrics and Gynaecology, Elkerliek Ziekenhuis, Helmond, 5707 HA The Netherlands; 14 Department of Obstetrics and Gynaecology, Rijnstate Ziekenhuis, Arnhem, 6815 AD The Netherlands; 15 Department of Obstetrics and Gynaecology, Maastricht Universitair Medisch Centrum +, Maastricht, 6229 HX The Netherlands; 16 Department of Obstetrics and Gynaecology, Noordwest Ziekenhuisgroep, Alkmaar, 1815 JD The Netherlands; 17 Department of Obstetrics and Gynaecology, Zaans Medisch Centrum, Zaandam, 1502 DV The Netherlands; 18 Department of Obstetrics and Gynaecology, Catharina Ziekenhuis, Eindhoven, 5623 EJ The Netherlands; 19 Department of Obstetrics and Gynaecology, Haga Ziekenhuis, Den Haag, 2545 AA The Netherlands; 20 Radiology and Nuclear Medicine, Amsterdam UMC location University of Amsterdam, Meibergdreef 9, Amsterdam, 1105 AZ Netherlands; 21 Department of Radiology, Imperial College Healthcare NHS Trust, Hammersmith Hospital, London, W12 0HS England; 22 Department of Obstetrics and Gynaecology, Monash University, Clayton, VIC 3168 Australia; 23 Aberdeen Centre for Women’s Health Research, School of Medicine, Medical Sciences and Nutrition, University of Aberdeen, Aberdeen, UK

**Keywords:** Infertility, Fallopian tubes, Fertility work-up, Tubal flushing, Hysterosalpingography (HSG), Oil-based contrast medium, Pregnancy, Live birth, Cost-effectiveness, Randomized controlled trial

## Abstract

**Background:**

In women with unexplained infertility, tubal flushing with oil-based contrast during hysterosalpingography leads to significantly more live births as compared to tubal flushing with water-based contrast during hysterosalpingography. However, it is unknown whether incorporating tubal flushing with oil-based contrast in the initial fertility work-up results to a reduced time to conception leading to live birth when compared to delayed tubal flushing that is performed six months after the initial fertility work-up. We also aim to evaluate the effectiveness of tubal flushing with oil-based contrast during hysterosalpingography versus no tubal flushing in the first six months of the study.

**Methods:**

This study will be an investigator-initiated, open-label, international, multicenter, randomized controlled trial with a planned economic analysis alongside the study. Infertile women between 18 and 39 years of age, who have an ovulatory cycle, who are at low risk for tubal pathology and have been advised expectant management for at least six months (based on the Hunault prediction score) will be included in this study. Eligible women will be randomly allocated (1:1) to immediate tubal flushing (intervention) versus delayed tubal flushing (control group) by using web-based block randomization stratified per study center. The primary outcome is time to conception leading to live birth with conception within twelve months after randomization. We assess the cumulative conception rate at six and twelve months as two co-primary outcomes. Secondary outcomes include ongoing pregnancy rate, live birth rate, miscarriage rate, ectopic pregnancy rate, number of complications, procedural pain score and cost-effectiveness. To demonstrate or refute a shorter time to pregnancy of three months with a power of 90%, a sample size of 554 women is calculated.

**Discussion:**

The H2Oil-timing study will provide insight into whether tubal flushing with oil-based contrast during hysterosalpingography should be incorporated in the initial fertility work-up in women with unexplained infertility as a therapeutic procedure. If this multicenter RCT shows that tubal flushing with oil-based contrast incorporated in the initial fertility work-up reduces time to conception and is a cost-effective strategy, the results may lead to adjustments of (inter)national guidelines and change clinical practice.

**Trial registration number:**

The study was prospectively registered in International Clinical Trials Registry Platform (Main ID: EUCTR2018-004153-24-NL).

**Supplementary Information:**

The online version contains supplementary material available at 10.1186/s12905-023-02385-1.

## Background

Infertility is defined as the inability to conceive within one year of unprotected intercourse and affects approximately one out of six couples [1]. In these couples fertility work-up can be performed to identify an underlying cause for infertility, which is found in approximately 70–85% of the cases. The most prevalent causes of infertility are ovulation disorders, tubal disease and male factor infertility. In the remaining part of the infertile couples the reason for infertility is unexplained [2, 3].

An essential component of the fertility work-up includes evaluating the risk for tubal pathology based on women’s medical history and Chlamydia IgG antibody test (CAT), and if indicated examining tubal patency through tubal patency testing [4, 5]. Hysterosalpingography (HSG) is typically the preferred method for tubal patency testing during the fertility work-up. Although HSG was introduced as a diagnostic test, the fertility enhancing effect of tubal flushing with oil-based contrast during HSG has been debated for decades [6–8]. In 2017, a large multicenter randomized controlled trial (RCT, H2Oil study) demonstrated that in women with unexplained infertility undergoing HSG with oil-based contrast this resulted in 11% more ongoing pregnancies compared to HSG with water-based contrast. The subsequent live birth rate was also significantly increased [9]. This fertility enhancing effect of oil-based contrast during HSG is confirmed in two recent meta-analyses [10, 11] and a Cochrane systematic review [12]. The latter included six RCTs comparing pregnancy rates within six months after tubal flushing with oil- versus water-based contrast during HSG (odds ratio (OR) 1.42; 95% confidence interval (CI) 1.10 to 1.85) and four RCTs comparing tubal flushing with oil-based contrast during HSG versus no tubal flushing (OR 3.54; 95% CI 2.08 to 6.02) [12].

The confirmation of the fertility-improving benefits of tubal flushing with oil-based contrast in women with unexplained infertility has expanded the role of HSG beyond its diagnostic function during fertility work-up. The next knowledge gap that needs to be addressed is determining the optimal timing for performing tubal flushing in relation to the fertility work-up considering its therapeutic role as well. In the Netherlands, the timing of start of fertility treatment for couples with unexplained infertility is based on their prognosis for natural conception within twelve months, and is estimated using the prognostic model of Hunault [13, 14]. In couples with a favorable prognosis, i.e. above 30%, expectant management is advised for a period of at least six months followed by intra-uterine insemination (IUI) in absence of the achievement of pregnancy. HSG is usually performed by the end of the period of expectant management. However, considering the therapeutic aspect of tubal flushing, an HSG performed during the fertility work-up instead of six months after the expectant management period might lead to a shortened time to conception.

The aim of this study is to evaluate the optimal timing for therapeutic flushing in couples with unexplained infertility. In this study we compare the effectiveness and cost-effectiveness of immediate tubal flushing with oil-based contrast during HSG incorporated in the fertility work-up compared to delayed tubal flushing that is performed six months after the fertility work-up is completed. We hypothesize that immediate tubal flushing (integrated in the fertility work-up) is a cost-effective strategy leading to a shorter time to pregnancy, more live births and less expensive and invasive fertility treatments compared to delayed tubal flushing.

## Methods

This study is an investigator-initiated, open label, multicenter, randomized controlled trial performed in the Netherlands and the United Kingdom. Participating hospitals include district, teaching and university hospitals, listed in appendix A. The study has granted ethical approval by the National Central Committee on Research involving Human Subjects (CCMO – NL 62838.029.18), by the Ethics committee of the Amsterdam UMC, location Vrije Universiteit Amsterdam (Ref. No. 2018.291, date 25th of July 2019) and by the boards of all participating hospitals. The study is also approved by the Research Ethics Committee London Harrow in the UK (Ref. No. 20/LO/0608, date 1st of July 2020). The first participant is enrolled on the 22nd of August 2019.

### Study population

In this study we will include women with unexplained infertility between 18 and 39 years of age, who have a regular ovulatory cycle (defined as eight or more spontaneous menstrual cycles per year), who are at low risk for tubal pathology and have been advised expectant management for at least six months. Women will not be included if they are at high risk for tubal pathology, defined as history of pelvic inflammatory disease, chlamydia infection, positive CAT, peritonitis, intestinal surgery, surgery of the Fallopian tubes and/or ovaries and endometriosis. Women are excluded if they have endocrine disorders (except for well managed hypothyroidism with thyroid-stimulating hormone (TSH) concentration < 2.5 mU/L) or iodine contrast medium allergy. Infertility is defined as the failure to achieve pregnancy after at least twelve months of unprotected intercourse. The male partner or sperm donor should have normal or mild impaired semen quality, defined as a pre-washed total motile sperm count (TMSC) above 3 × 10^6^ spermatozoa per milliliter. Inventory of the medical history, physical examination and blood tests will be done during the fertility work-up, which determine if expectant management is advised.

### Recruitment, randomization and blinding

Eligible women will be identified by their clinician during the fertility work-up. Counselling for the study will be done by a trained research staff member and all women will receive written information. Couples will be given a minimum of two days to consider participation. Those women who agree to participate will be asked to sign a written consent form. After informed consent, women will be randomly allocated to the intervention group (immediate HSG) or control group (delayed HSG). Randomization will be performed via the web-based application Castor Electronic Date Capture (EDC, Amsterdam, the Netherlands) with the use of permuted block design stratified per center (block size: 4, 6 or 8). Owing to the nature of the intervention and since our primary outcome of time to conception leading to live birth is objective, the trial is not blinded. After randomization the data will be collected in an electronic case report file (eCRF), using Castor EDC. All study procedures will be performed by clinicians and other research employees trained according to the Good Clinical Practice guidelines.

### Outcomes

The primary outcome is time to conception leading to live birth, with the first day of the last menstrual bleeding before a positive pregnancy test within 12 months after randomization. We will assess the presence of this endpoint also at six months after randomization as a co-primary outcome. All outcomes are listed and described in Table 1.

**Table 1:** Outcomes and descriptions

**Primary outcome**	**Definition/measurement**
Time to conception leading to live birth measured at 6 months	Calculated from the first day of the last menstrual bleeding before a positive pregnancy test
Time to conception leading to live birth measured at 12 months	Calculated from the first day of the last menstrual bleeding before a positive pregnancy test
**Secondary outcome**	**Definition/measurement**
Biochemical pregnancy	A positive pregnancy test or serum HCG-level greater than 5 IU/L
Clinical pregnancy	Gestational sac visible on ultrasonography
Ongoing pregnancy	Positive heartbeat at 12 weeks of pregnancy on ultrasonography
Miscarriage	Non-vitality on ultrasound or spontaneous loss of pregnancy
Ectopic pregnancy	No intrauterine gestational sac with an ectopic gestational sac and/or persistent serum HCG levels
Multiple pregnancy	Pregnancy of two or more fetuses
Complications after HSG	All adverse events occurring within one month after HSG procedure, for example pelvic infection, intravasation and allergic reaction
Level of pain during HSG	Reported on VAS (scores: 0.0 to 10.0 cm)
Thyroid function after HSG	Serum FT4 and TSH one month after HSG
Used fertility treatments	IUI, IVF, ICSI
Pregnancy complications	E.g. pregnancy induced hypertension, gestational diabetes, still birth
Pregnancy outcomes	E.g. gestational age, pre-term birth, birth weight
Cost-effectiveness	Effectiveness: time to conception leading to live birth Costs: Healthcare perspective: costs for HSG and fertility treatments Societal perspective: healthcare costs, loss of productivity costs and patient costs (care services paid for by the patients themselves) using iMCQ and iPCQ

HCG: human chorionic gonadotropin, HSG: hysterosalpingography. VAS: Visual Analogue Scale, FT4: free thyroxine, TSH: thyroid-stimulating hormone, IUI: intrauterine insemination, IVF: in vitro fertilization, ICSI: intracytoplasmatic sperm injection, iMCQ: Medical Consumption Questionnaire, iPCQ: Productivity Cost Questionnaire

### Description of intervention

Women allocated to immediate HSG will undergo their HSG preferably in the month following randomization and will subsequently have expectant management for at least six months if the HSG findings are normal.

### Description of control

Women allocated to six-months delayed HSG will have expectant management for six months followed by undergoing HSG, if an ongoing pregnancy was not achieved in the meantime.

### HSG procedure

In both arms, tubal flushing during HSG will be performed according to the local protocol of each participating center during the follicular phase of the menstrual cycle after complete cessation of menstrual bleeding. The HSG will be undertaken using oil-based contrast (Lipiodol® Ultra Fluid, Guerbet, Villepinte, France). Prior to the HSG women will be asked to fill out the modified Amsterdam Preoperative Anxiety and Information Scale (APAIS) questionnaire to assess their pre-procedural anxiety level [15]. During the HSG, oil-based contrast medium will be infused into the uterine cavity through a special HSG-balloon catheter, a cervical vacuum cup or a metal/acorn cannula. The maximum recommended volume of the contrast medium is 15ml to limit women’s exposure to iodine. Batch number and expiration date of the used contrast medium will be reported for drug accountability. During infusion of the contrast under fluoroscopic control four to six radiographs or images will be taken to evaluate the filling and shape of the uterus and the patency of the Fallopian tubes. Immediately after the procedure women’s pain experience will be evaluated, using the Visual Analogue Scale (VAS) ranging from 0.0 to 10.0 cm. According to local protocols, in some cases a final radiograph may be taken 20 to 30 min later to evaluate the dissemination of the contrast in the abdominal cavity. Antibiotic therapy (e.g. Doxycylin 200 mg BDS for 7 days) will be prescribed in case of suspected intra-abdominal adhesions or hydrosalpinx, according to the local protocols of the participating centers. The procedure will be immediately discontinued if an allergic reaction occurs or intravasation of the contrast medium is noticed on the radiographs to minimize to risk of oil-embolism. The HSG radiographs and images will be evaluated by a gynecologist and/or radiologist according to the local protocol of the participating centers.

### Follow-up

For the primary study, we will follow-up women for twelve months, with conception rates assessed six and twelve months after randomization. For each conception within twelve months after randomization, we will assess whether this conception leads to live birth beyond the twelve months window. Follow-up outcome data will be extracted from women’s medical records and/or by sending a digital questionnaire send to them through Castor EDC. The follow-up data include information about fertility treatments (used to calculate direct medical costs), (ongoing) pregnancy(ies) and delivery if applicable. At six and twelve months after randomization women will receive a digital questionnaire send through Castor EDC to explore indirect costs from a societal perspective during the preceding six months (iMCQ [16] and iPCQ [17]). Table 2 provides an overview of the study activities. For a secondary analysis, we will follow women until 36 months after randomization.

**Table 2:** SPIRIT schedule of enrollment, intervention and assessments

	Study period					
	Enrollment	Allocation	Post-allocation			
TIMEPOINT	-t_1_	t_0_	t_1_	t_2_	t_3_	t_4_
ENROLLMENT:						
Eligibility screening	X					
Informed consent	X					
Allocation		X				
INTERVENTIONS:						
Immediate oil-HSG			X			
Delayed oil-HSG				X		
ASSESSMENTS:						
Baseline characteristics		X				
APAIS questionnaire*			X or X			
Pain score*			X or X			
HSG procedure, results and complications*			X or X			
iMCQ				X	X	
iPCQ				X	X	
Fertility treatment					X	
Pregnancy yes/no				X	X	
Ongoing pregnancy outcome						X

*Depending on the allocated intervention. -t_1_: prior to allocation, t_0_: at allocation, t_1_: first menstrual cycle after allocation, t_2_: 6 months after allocation, t_3_: 12 months after allocation, t_4_: 2 months post-partum

### Sample size

The time-to-event method was used for the sample size calculation, based on the study of Dreyer et al. [9]. The expected cumulative live birth rate in the control group is 53% with a median time to event of 11 months in the control group. A two-sided log rank test with an overall sample size of 554 subjects achieves 90% power at a 0.05 significance level to or refute a statistically significant effect with a hazard radio of 1.39. It is assumed that 0.32% of the couples switch from one group to the other group. Sample size calculation was done using PASS 15.0.6 (NCSS Statistical Software LCC, Utah, USA).

### Statistical analyses

The primary analysis will be performed according to the intention-to-treat principle. Continuous variables that are normally distributed will be summarized as means with standard deviation and, if not normally distributed, as medians with an inter-quartile range. Dichotomous data will be reported as proportions with percentages. We will summarize recruitment numbers, lost to follow-up, protocol violations and other relevant data.

The effectiveness of a fertility work-up with a strategy of immediate tubal flushing during HSG versus a strategy of six-month delayed tubal flushing during HSG will be expressed as a rate of live birth with corresponding 95% confidence intervals. Time to pregnancy will be compared in both groups using Cox Proportional-hazards analysis.

Dichotomous outcomes will be analyzed using either modified Poisson regression or logistic regression as appropriate. Continuous outcomes which are normally distributed will be analyzed using linear regression and if outcomes are non-normally distributed negative binomial regression will be used. Although p-values will be reported, the focus will be on providing Risk Ratios for effectiveness with 95% confidence intervals around them as these are more useful in interpreting the findings of the trial.

For losses to follow-up, protocol violations and missing data, we will attempt worst-case scenario analysis to explore the effect of these factors on the trial findings. As a sensitivity analysis, we will explore the effects of missing data on the trial findings using imputation techniques.

Apart from the analysis for the primary outcome – time to conception within twelve months leading to live birth - we will also perform an analysis comparing the time to conception and number of conceptions within 6 months after randomization that lead to live birth. This will allow us to assess the effect of immediate flushing with oil-based contrast as compared to no flushing.

Apart from the intention-to treat analysis, we planned a per protocol analysis. We will write a separate statistical analysis plan that will specify the details of this analysis. SPSS software (IBM) and R software (R Project for Statistical Computing) will be used for all statistical analyses.

### Economic evaluation

The economic evaluation will be performed alongside the clinical trial. The aim of the economic evaluation is to relate the incremental costs of immediate tubal flushing at HSG with oil-based contrast during the initial fertility work-up (intervention group) in comparison with delayed tubal flushing at HSG with oil-based contrast six months after completing the fertility work-up (control group) to the incremental health effects. The cost-effectiveness analysis will be performed from a societal and healthcare perspective according to Dutch guidelines with a time horizon of twelve months [18]. Costs will be measured from a societal perspective using web-based questionnaires consisting of the iPCQ and iMCQ at six and twelve months of follow-up. Cost categories that will be included are: healthcare costs, loss of productivity costs and patient costs (care services paid for by the patients themselves). The healthcare costs include the costs made to achieve conception leading to a live birth and costs for pregnancy and delivery. The statistical analyses will be done according to the intention-to-treat principle. Incremental cost-effectiveness ratios (ICERs) will be calculated by dividing the difference in the mean total costs between the treatment groups by the difference in mean effect between the treatment groups.

Subsequently, a budget impact analysis will be conducted. The data from the clinical study and the cost-effectiveness analysis regarding the differences in costs and health outcomes will be combined with national prevalence and incidence data to extrapolate the findings to a time horizon of 5 years. The economic analysis will be reported in a separate paper.

### Data management and monitoring

Study data will be collected in the web-based application Castor EDC, using allocated, anonymous randomization numbers for each participant. Only local investigators have access to the key document with the linkage of the randomization number and personal data.

An interim analysis is not planned. Suspected unexpected serious adverse reactions (SUSARs) and severe adverse events (SAEs) will be reported to the sponsor and the sponsor will report them through the Dutch web portal ToetsingOnline to the accredited Medical Ethics Committee (IRB) of Amsterdam UMC, location Vrije Universiteit Amsterdam. We will report SAEs which occur within one month after the HSG procedure and congenital anomalies and birth defects. All adverse events (AEs) directly related to the HSG procedure will be reported and followed until they have abated or until a stable situation has been reached. Yearly, a safety report will be submitted to the IRB of Amsterdam UMC, location Vrije Universiteit Amsterdam, which consists of all the suspected SAEs and a report concerning the safety of the subjects.

A data safety monitoring board will not be installed since the product and intervention used in this study are registered for the given indication and have been used in clinical practice for decades.

Monitoring will be performed by an independent monitor (Clinical Monitoring Center, Amsterdam UMC) according to Good Clinical Practice guidelines. The monitor will have access to the data and source documents of the trial. Signed informed consent forms are stored in the local participating centers, all forms and study data will be archived for at least 25 years in the participating centers according to national regulations.

## Discussion

The role of HSG during the fertility work-up and the subsequent treatment advice in women with unexplained infertility varies between guidelines worldwide. Table 3 represents an overview of different international guidelines for tubal patency testing and treatment for couples with unexplained infertility. In these guidelines HSG is currently used as a diagnostic tool for assessing tubal patency, without considering the established fertility enhancing effect of tubal flushing during HSG. Additionally, these guidelines do not specify the type of contrast to be used during HSG.

**Table 3:** Overview of the guidelines for unexplained infertility in the United Stated, the United Kingdom and the Netherlands

Guideline	Tubal patency test		Treatment
	Low risk for tubal pathology	High risk for tubal pathology	
ASRM, the United States (5)	HSG	HSG	3–4 Cycles of hormonal stimulated IUI before starting IVF
NICE, the United Kingdom (4)	HSG	Laparoscopy	In total 2 years of regular unprotected intercourse before starting IVF
NVOG, the Netherlands (6)	No tubal patency test	HSG	Favorable chance to conceive (> 30%, based on the Hunault prediction score(14)) within 1 year: 6–12 months expected management, before start of 6 cycles hormonal stimulated IUI followed by IVF. Unfavorable chance (< 30%): 6 hormonally stimulated IUI cycles followed by IVF

ASRM: American Society for Reproductive Medicine, NICE: National Institute for Health and Care Excellence, NVOG: the Netherlands Society of Obstetrics and Gynaecology Global Network, IUI: intrauterine insemination, IVF: in vitro fertilization

The H2Oil-timing study will provide insight into whether HSG with oil-based contrast should be incorporated as a therapeutic procedure during the initial fertility work-up in women with unexplained infertility. If incorporating HSG with oil-based contrast as standard part of the fertility work-up leads to a shorter time to conception and higher live birth rate, fewer couples will need costly fertility treatments. This is particularly significant since fertility treatments are known to cause substantial psychological burden to infertile couples[19, 20].

There is limited literature on the therapeutic role of HSG during fertility work-up in women with unexplained infertility. While two randomized controlled trials with small sample sizes have demonstrated a positive effect of tubal flushing during HSG with oil-based contrast on pregnancy rates compared to no tubal flushing in women with unexplained infertility, information on live birth rates and time to pregnancy was not investigated [21, 22]. Recently, a Dutch controlled cohort study compared pregnancy rates after an extensive fertility work-up and a concise (standard) fertility work-up [23]. The concise fertility work-up included inventory of the medical history, physical examination, transvaginal ultrasound and semen analysis. Other tests were performed if indicated. Extensive work-up included the elements of the concise work-up plus cycle monitoring, Chlamydia antibody determination, postcoital test and tubal patency testing by HSG. After follow-up of twelve months the ongoing pregnancy rate for couples who underwent the extensive fertility work-up was significantly higher compared to the couples who underwent the concise fertility work-up (59% vs. 47%; p < 0.001). HSG was performed significantly more often during the extensive fertility work-up than during the concise fertility work-up (42% vs. 33%; p < 0.001) and HSG was often performed within the first six months after start of the fertility work-up. In couples who underwent the extensive fertility work-up the time to pregnancy was shortened as well. These findings support our hypothesis that immediate HSG incorporated in the fertility work-up may improve fertility outcomes [23].

Worldwide health care costs are increasing [24]. When incorporating HSG with oil-based contrast as standard part of fertility work-up the additional costs need to be considered as well. Therefore, we will perform a cost-effectiveness analysis alongside the proposed study, considering the costs from a healthcare (costs for tubal patency testing and for fertility treatments) and societal perspective. The costs from a societal perspective will be estimated using validated questionnaires (iMCQ and iPCQ [16, 17]) to assess absenteeism, productivity loss and medical consumption.

As shown in Table 3, contrary to elsewhere, initiation of active fertility treatment for couples with unexplained infertility in the Netherlands is based on their prognosis for natural conception (using the prognostic model of Hunault [14]). Shingshetty et al. emphasizes the importance of a prognosis-based approach to couples with unexplained infertility to find balance between overtreatment and give access to treatment for those who benefit from it [25]. When interpreting the results of the H2Oil-timing, its consideration is required that the results of this study might not be directly applicable to guidelines which do not distinguish between a favorable and non-favorable prognosis to conceive naturally.

The safety of HSG with oil-based contrast is extensively described in a recent systematic review including all the published evidence since the 1920’s [26]. The most frequently reported complications after HSG with oil-based contrast are intravasation and infection. Intravasation occurred significantly more frequent after the use of oil-based contrast compared to the use of water-based contrast (3% vs. 2%; OR 5.05 95% CI 2.3–11.2; p < 0.0001), without cases of serious lasting consequences. To minimize the risk of formation of oil-embolism in the H2Oil-timing study, the HSG procedure will be immediately discontinued if intravasation is noticed. The incidence of pelvic infection after 1960 was 0.6% after oil-based contrast (95% CI 0.2-1.0) compared to 0.4% (95% CI 0.0-7.3) in the water-based group [26]. According to the recent published Cochrane systematic review it is uncertain whether tubal flushing with oil-based contrast increases the risk of infection due to very low quality of evidence [12]. In the H2Oil-timing study antibiotic therapy will be prescribed in cases of suspected intra-abdominal adhesions or hydrosalpinges. In the literature, concerns about maternal and neonatal thyroid dysfunction after HSG with oil-based contrast are reported as well. Several studies have shown that an iodine excess in urine was detected in the majority of the women after HSG with oil-based contrast which lasted for at least 12 to 24 weeks with a peak concentration by four weeks, leading to a prevalence of subclinical hypothyroidism of approximately 20% [27, 28]. Mekaru et al. showed that women with subclinical hypothyroidism before HSG were more at risk to develop hypothyroidism after HSG, compared to euthyroid women [29]. It is reassuring that recent studies have shown that preconceptional exposure to oil-based contrast during HSG did not increase the risk for thyroid dysfunction in offspring [27, 30, 31]. In the H2Oil-timing study women with thyroid dysfunction can only be included if their TSH serum concentration is below 2.5 mU/L, to prevent further exacerbation of the thyroid dysfunction.

In conclusion, if this study shows that tubal flushing with oil-based contrast incorporated during the initial fertility work-up reduces time to conception and is a cost-effective strategy, the results may lead to adjustments of (inter)national guidelines and change practice.

### Electronic supplementary material

Below is the link to the electronic supplementary material.

Supplementary Material 1: Trial registration data set.

Supplementary Material 2: List of participating sites.

## Data Availability

This manuscript is a study protocol. Datasets generated during the study will be available and published along with the results of the study.

## List of abbreviations

AE: Adverse Event
APAIS: Amsterdam Preoperative Anxiety and Information Scale
ASRM: American Society for Reproductive
CAT: Chlamydia IgG Antibody Test
CI: Confidence Interval
CRF: Case Report File
EDC: Electronic Date Capture
FT4: Free thyroxine
HCG: Human Chorionic Gonadotropin
HSG: Hysterosalpingography
ICSI: Intracytoplasmatic Sperm Injection
ICER: Increment Cost-Effectiveness Ratio
IRB: Institutional Review Board
IUI: Intra Uterine Insemination
iMCQ: IMTA Medical Consumption Questionnaire
iPCQ: IMTA Productivity Cost Questionnaire
IRB: Institutional Review Board
ITT: Intention to Treat
IVF: In Vitro Fertilization
NICE: National Institute for Healthcare and Excellence
NVOG: The Netherlands Society of Obstetrics and Gynaecology Global Network
OR: Odds Ratio
RCT: Randomized controlled trial
SAE: Severe Adverse Event
SUSAR: Suspected Unexpected Serious Adverse Reaction
TMSC: Total Motile Sperm Count
TSH: Thyroid Stimulation Hormone
VAS: Visual Analogue Scale
