# Correction: The effectiveness of immediate versus delayed tubal flushing with oil-based contrast in women with unexplained infertility (H2Oil-timing study): study protocol of a randomized controlled trial

**DOI:** 10.1186/s12905-023-02574-y

**Published:** 2023-08-10

**Authors:** D. Kamphuis, K. Rosielle, N. van Welie, I. Roest, A. J. C. M. van Dongen, E. A. Brinkhuis, P. Bourdrez, A. Mozes, H. R. Verhoeve, D. P. van der Ham, F. P. J. M. Vrouenraets, J. J. Risseeuw, T. van de Laar, F. Janse, J. E. den Hartog, M. de Hundt, A. B. Hooker, A. G. Huppelschoten, Q. D. Pieterse, M. Y. Bongers, J. Stoker, C. A. M. Koks, C. B. Lambalk, A. Hemingway, W. Li, B. W. J. Mol, K. Dreyer, V. Mijatovic

**Affiliations:** 1 Department of Reproductive Medicine, Amsterdam UMC Location Vrije Universiteit Amsterdam, De Boelelaan 1117, 1081HV, Amsterdam, 1081 HV The Netherlands; 2 Amsterdam Reproduction and Development Research Institute, Amsterdam, The Netherlands; 3 Department of Obstetrics and Gynaecology, OLVG, Amsterdam, 1091 AC The Netherlands; 4 Department of Obstetrics and Gynaecology, Máxima Medisch Centrum, Veldhoven, Eindhoven, 4600 DB The Netherlands; 5 Grow Research School for Oncology and Reproduction, Maastricht University, Universiteitssingel 40, Maastricht, 6229 ER The Netherlands; 6 Department of Obstetrics and Gynaecology, Ziekenhuis Gelderse Vallei, Ede, 6716 RP The Netherlands; 7 Department of Obstetrics and Gynaecology, Meander Medisch Centrum, Amersfoort, 3813 TZ The Netherlands; 8 Department of Obstetrics and Gynaecology, VieCuri Medisch Centrum, Venlo, 5912 BL The Netherlands; 9 Department of Obstetrics and Gynaecology, Ziekenhuis Amstelland, Amstelveen, 1186 AM The Netherlands; 10 Department of Obstetrics and Gynaecology, Martini Ziekenhuis, Groningen, 9728 NT The Netherlands; 11 Department of Obstetrics and Gynaecology, Zuyderland Medisch Centrum, Heerlen, 6419 PC The Netherlands; 12 Department of Obstetrics and Gynaecology, St. Jansdal Ziekenhuis, Harderwijk, 3844 DG The Netherlands; 13 Department of Obstetrics and Gynaecology, Elkerliek Ziekenhuis, Helmond, 5707 HA The Netherlands; 14 Department of Obstetrics and Gynaecology, Rijnstate Ziekenhuis, Arnhem, 6815 AD The Netherlands; 15 Department of Obstetrics and Gynaecology, Maastricht Universitair Medisch Centrum +, Maastricht, 6229 HX The Netherlands; 16 Department of Obstetrics and Gynaecology, Noordwest Ziekenhuisgroep, Alkmaar, 1815 JD The Netherlands; 17 Department of Obstetrics and Gynaecology, Zaans Medisch Centrum, Zaandam, 1502 DV The Netherlands; 18 Department of Obstetrics and Gynaecology, Catharina Ziekenhuis, Eindhoven, 5623 EJ The Netherlands; 19 Department of Obstetrics and Gynaecology, Haga Ziekenhuis, Den Haag, 2545 AA The Netherlands; 20 Radiology and Nuclear Medicine, Amsterdam UMC Location University of Amsterdam, Meibergdreef 9, 1105 AZ Amsterdam, Netherlands; 21 Department of Radiology, Imperial College Healthcare NHS Trust, Hammersmith Hospital, London, W12 0HS England; 22 Department of Obstetrics and Gynaecology, Monash University, Clayton, VIC 3168 Australia; 23 Aberdeen Centre for Women’s Health Research, School of Medicine, Medical Sciences and Nutrition, University of Aberdeen, Aberdeen, UK

**Correction: BMC Women’s Health 23, 233 (2023)**

**https://doi.org/10.1186/s12905-023-02385-1**

Following publication of the original article [1], in this article the "Trial registration number" under the abstract has been updated and the same will read as follow:

Trial registration number: The study was prospectively registered in International Clinical Trials Registry Platform (Main ID: EUCTR2018-004153-24-NL).

The original article has been corrected.
