# Supplementary material for: The effectiveness of immediate versus delayed tubal flushing with oil-based contrast in women with unexplained infertility (H2Oil-timing study): study protocol of a randomized controlled trial
Source: BMC Womens Health. 2023 May 6;23:233. doi: 10.1186/s12905-023-02385-1 (PMC10164300; doi:10.1186/s12905-023-02385-1)
Supplement: Supplementary file 1 — Supplementary Material 1: Trial registration data set. [file 12905_2023_2385_MOESM1_ESM.docx]

**Table S1. Trial registration data set.**

| Data category | Information |
| --- | --- |
| Primary registry and trial identifying number | International Clinical Trials Registry Platform, EUCTR2018-004153-24-NL |
| Date of registration in primary registry | 30/07/2019 |
| Secondary identifying numbers | Clinicaltrial.gov identifier: NCT05608590 |
| Source of monetary of material support | ZonMw, the Netherlands  Guerbet |
| Primary sponsor | Stichting VUmc |
| Secondary sponsor | N/A |
| Contact for public queries | DK, VM  [d.kamphuis@amsterdamumc.nl](mailto:d.kamphuis@amsterdamumc.nl); +3120 444 4567  De Boelelaan 1117, 1081 HV Amsterdam, the Netherlands |
| Contact for scientific queries | Prof. dr. V. Mijatovic, [mijatovic@amsterdamumc.nl](mailto:mijatovic@amsterdamumc.nl); +3120 444 4567  De Boelelaan 1117 ,1081 HV Amsterdam, the Netherlands  Study team  [H2Olie-timing@amsterdamumc.nl](mailto:H2Olie-timing@amsterdamumc.nl); +3120 444 4567  De Boelelaan 1117, 1081 HV Amsterdam, the Netherlands |
| Public title | The effectiveness of immediate versus delayed tubal flushing with oil-based contrast in women with unexplained infertility (H2Oil-timing study): study protocol of a randomized controlled trial |
| Scientific title | The effectiveness of immediate versus delayed tubal flushing with oil-based contrast in women with unexplained infertility (H2Oil-timing study): study protocol of a randomized controlled trial |
| Countries of recruitment | The Netherlands, the United Kingdom |
| Health condition(s) or problem(s) studied | Infertility |
| Intervention | Intervention group: immediate tubal flushing with oil-based contrast fluid (Lipiodol Ultra Fluide, Guerbet, Villepinte, France) during HSG as part of the initial fertility work-up  Control group: six months delayed tubal flushing with oil-based contrast fluid (Lipiodol Ultra Fluide, Guerbet, Villepinte, France) during HSG |
| Key inclusion and exclusion criteria | Inclusion criteria:   - - Women between 18-39 years of age - Spontaneous menstrual cycle - Perceived low risk for tubal pathology - Undergoing fertility work-up   Exclusion criteria:  - Women with known endocrine disorders (e.g. the polycystic ovary syndrome, diabetes, hyperthyroidism and hyperprolactinemia. Except for well managed hypothyroidism with TSH between 0.3 and 2.5mIU/l) - Ovulation disorders defined as less than eight menstrual cycles per year - Iodine allergy - Male subfertility defined as a post-wash total motile sperm count < 1 x10^6 spermatozoa/ml - Not willing or able to sign the consent form |
| Study type | Interventional  Allocation: randomized intervention model, not blinded, parallel  Randomization: online using a permuted block design with blocks size 4, 6 and 8, stratified for inclusion site  Primary purpose: treatment  Phase: post market phase |
| Date of first enrollment | August 22^th^ 2019 |
| Sample size | Number of participants planned: 554  Current inclusion number: 473 |
| Recruitment status | Recruiting |
| Primary outcome(s) | time to conception leading to live birth, calculated from the first day of the last menstrual bleeding before a positive pregnancy test measured at 6 and 12 months after randomization |
| Key secondary outcome(s) | Other pregnancy outcomes within 6 and 12 months of randomization: biochemical pregnancy (positive pregnancy test or elevated HCG level), clinical pregnancy (ultrasound confirmed intrauterine gestational sac), ongoing pregnancy (positive fetal heartbeat on ultrasound examination after 12 weeks of gestation), miscarriage (loss clinical or ongoing pregnancy or diagnosis of a pregnancy without positive fetal heartbeat before 12 weeks gestation), ectopic pregnancy (ultrasound or surgically confirmed extra uterine pregnancy), multiple pregnancy (two or more fetuses). Pregnancy complications, complications of HSG such as intravasation, infection and hypo- or hyperthyroidism, and a cost-effectiveness analysis will also be part of the secondary outcomes. Procedural pain measured using a VAS-ruler within 10 minutes of the procedure |
| Ethics review | Status: approved  Date of approval: July 25th 2019  Ethics committee: Medisch Ethische Toetsingscommissie, +3120 444 5585, [metc@vumc.nl](mailto:metc@vumc.nl) |
| Completion data | N/A |
| Summary results | N/A |
| IPD sharing statement | The completed study database will be available on an online repository for further research upon reasonable request after an embargo period. |
