# Supplementary material for: The effectiveness of immediate versus delayed tubal flushing with oil-based contrast in women with unexplained infertility (H2Oil-timing study): study protocol of a randomized controlled trial
Source: BMC Womens Health. 2023 May 6;23:233. doi: 10.1186/s12905-023-02385-1 (PMC10164300; doi:10.1186/s12905-023-02385-1)
Supplement: Supplementary file 2 — Supplementary Material 2: List of participating sites. [file 12905_2023_2385_MOESM2_ESM.docx]

# Appendix A

List of participating sites (30th December 2022)

The Netherlands

Amstelland Hospital, Amstelveen – PI drs. A. Mozes

Amsterdam UMC, location Vrije Universiteit, Amsterdam – PI Prof. dr. V. Mijatovic

Catharina Hospital, Eindhoven – PI dr. A.G. Huppelschoten

Elkerliek Hospital, Helmond – PI dr. J. Penninx

Gelderse Vallei Hospital, Ede – PI dr. A.J.C.M. van Dongen

Haga Hospital, Den Haag – PI dr. Q.D. Pieterse

Maastricht University Medical Center, Maastricht – PI dr. J.E. den Hartog

Martini Hospital, Groningen – PI dr. D.P. van der Ham

Meander Medical Center, Amersfoort – PI drs. E.A. Brinkhuis

North West Hospital Group, Alkmaar – PI dr. M. de Hundt

OLVG, Amsterdam – PI dr. H.R. Verhoeve

Rijnstate Hospital, Arnhem – PI dr. F. Janse

St. Jansdal Hospital, Hardewijk – PI drs. J.J. Risseeuw

Viecuri, Venlo – PI drs. P. Bourdrez

Zaans Medical Center, Zaandam – PI drs. A.B. Hooker

Zuyderland Medical Center, Heerlen – PI drs. F.P.J.M. Vrouenraets

United Kingdom

Imperial College NHS Healthcare Trust, London, PI prof. dr. Hemingway
